# Muscarinic Depolarization of Layer II Neurons of the Parasubiculum

**DOI:** 10.1371/journal.pone.0058901

**Published:** 2013-03-08

**Authors:** Stephen D. Glasgow, C. Andrew Chapman

**Affiliations:** Center for Studies in Behavioral Neurobiology, Department of Psychology, Concordia University, Montréal, Québec, Canada; University of North Dakota, United States of America

## Abstract

The parasubiculum (PaS) is a component of the hippocampal formation that sends its major output to layer II of the entorhinal cortex. The PaS receives strong cholinergic innervation from the basal forebrain that is likely to modulate neuronal excitability and contribute to theta-frequency network activity. The present study used whole cell current- and voltage-clamp recordings to determine the effects of cholinergic receptor activation on layer II PaS neurons. Bath application of carbachol (CCh; 10–50 µM) resulted in a dose-dependent depolarization of morphologically-identified layer II stellate and pyramidal cells that was not prevented by blockade of excitatory and inhibitory synaptic inputs. Bath application of the M_1_ receptor antagonist pirenzepine (1 µM), but not the M_2_-preferring antagonist methoctramine (1 µM), blocked the depolarization, suggesting that it is dependent on M_1_ receptors. Voltage-clamp experiments using ramped voltage commands showed that CCh resulted in the gradual development of an inward current that was partially blocked by concurrent application of the selective Kv7.2/3 channel antagonist XE-991, which inhibits the muscarine-dependent K^+^ current *I*_M_. The remaining inward current also reversed near E_K_ and was inhibited by the K^+^ channel blocker Ba^2+^, suggesting that M_1_ receptor activation attenuates both *I*_M_ as well as an additional K^+^ current. The additional K^+^ current showed rectification at depolarized voltages, similar to K^+^ conductances mediated by Kir 2.3 channels. The cholinergic depolarization of layer II PaS neurons therefore appears to occur through M_1_-mediated effects on *I*_M_ as well as an additional K^+^ conductance.

## Introduction

Recent evidence suggests that the parasubiculum (PaS), which is a major component of the subicular complex, plays an important role within the brain navigational system [1], [2], [3]. The PaS receives numerous cortical and subcortical inputs, including substantial projections from the CA1 region of the hippocampus, the anterior thalamus, and medial septum [4], [5], [6]. Layer II parasubicular neurons, in turn, send projections almost exclusively to layer II of the entorhinal cortex which contains the cells of origin of the perforant path [6], [7], [8], [9]. Layer II of the entorhinal cortex serves as an interface between sensory associational cortices and the hippocampus, and inputs from the PaS may therefore modulate how the entorhinal cortex mediates the transfer of highly processed sensory information to the hippocampus [10].

We have recently shown that atropine-sensitive theta-frequency LFP activity is generated locally within the superficial layers of the PaS, and that layer II PaS neurons display theta-frequency oscillations in membrane potential at near-threshold voltages [11], [12]. Septal cholinergic inputs may therefore help to generate membrane potential oscillations by depolarizing PaS neurons to near-threshold voltages [13]. In other regions of the hippocampal formation, acetylcholine is known to induce prominent changes in overall neuronal excitability, including alterations in spike properties, increases in input resistances, and sustained membrane potential depolarization via alterations in various conductances [14], [15], [16]. Early work in hippocampal neurons demonstrated that activation of muscarinic receptors results in the inhibition of K^+^ conductances, including the muscarine-sensitive K^+^ current *I*_M_ and a leak K^+^ current [17], [18]. In contrast, muscarinic depolarization of principal neurons of the prefrontal and entorhinal cortices has been attributed primarily to activation of a Ca^2+^-modulated nonselective cationic current [19], [20]. Cholinergic modulation of principal cell types in retrohippocampal cortices is likely to play an important role in encoding and retrieval processes associated with these regions [16], [21], and therefore it is crucial to understand how neuromodulators such as acetylcholine modulate the basic cellular properties of parasubicular neurons.

The present study was aimed at determining the ionic conductances responsible for changes in membrane potential following muscarinic receptor activation in morphologically-identified layer II neurons of the PaS. Whole cell current- and voltage-clamp recordings in acute brain slices were used to characterize changes in membrane potential and firing properties of PaS neurons in response to the cholinergic agonist carbachol. Cholinergic receptor activation was found to modulate neuronal excitability via actions on M_1_ receptors, and that the depolarization of PaS neurons by carbachol was found to be dependent on suppression of *I*_M_ as well as an additional K^+^ conductance that is likely mediated by Kir2 channels.

## Materials and Methods

The methods used for slice preparation were conducted in accordance with the guidelines of the Canadian Council on Animal Care, and were approved by the Concordia University Animal Research Ethics committee.

### Slice preparation

Acute brain slices were obtained from 4 to 6 week old Long-Evans male rats (Charles River Laboratories, Saint-Constant, QC). Rats were deeply anesthetized using halothane and decapitated. The brain was quickly extracted and submerged in ACSF (4 °C) containing (in mM): 124 NaCl, 5 KCl, 1.25 NaH_2_PO_4_, 2 MgSO_4_, 2 CaCl_2_, 26 NaHCO_3_, and 10 dextrose saturated with 95% O_2_ and 5% CO_2_ (pH ∼7.3; 300–310 mOsm). Horizontal hippocampal slices (300 µM thick) containing the PaS were cut using a vibratome (WPI, Vibroslice NVSL, Sarasota, FL, USA), and allowed to recover for ∼1.5 h at room temperature. Individual slices were transferred to a recording chamber, and superfused with oxygenated room temperature ACSF (∼22–24 °C) at a rate of 1.5–2.0 ml/min to reduce metabolic demand on the cells. Slice landmarks, such as the characteristic broadening of layer II/III of the PaS compared to the relatively compact superficial layers of the adjacent presubiculum and medial entorhinal cortex, as well as the location of the angular bundle, were used to identify the location of the PaS [11], [12], [22]. Individual PaS neurons were visualized using an upright microscope (Leica, DM-LFS, Concord, ON, CA) equipped with a long-range (40x) water immersion objective using differential interference contrast optics, and a near-infrared camera (COHU, Series 4990, Poway, CA, USA).

### Electrophysiological recordings and data analysis

Somatic whole-cell recordings were obtained using patch recording pipettes (4–8 MΩ) containing (in mM): 140 K-gluconate, 5 NaCl, 2 MgCl_2_, 10 N-2-hydroxyethylpiperazine-N′-2-ethanesulfonic acid (HEPES), 0.5 ethylene glyco-bis (β-aminoethyl ether)-N,N,N′,N′-tetraacetic acid (EGTA), 2 ATP-Tris, 0.4 GTP-Tris (pH adjusted to 7.20–7.26 using KOH; 270–280 mOsm). Biocytin (0.1%) was added in some experiments to allow morphological identification of neurons. Pipettes were prepared from borosilicate glass capillaries (1.0 mm OD, 0.5 mm ID) using a horizontal puller (Sutter Instr., P-97, Novato, CA, USA). After contacting the soma of the target cell, gentle suction was applied in voltage-clamp mode to obtain a tight seal (>1 GΩ), and strong suction was then applied to obtain whole-cell configuration. Cells were allowed to recover for ∼5 min before recordings were taken. An Axoclamp 200B amplifier (Molecular Devices, Sunnyvale, CA, USA) was used for current-clamp and voltage-clamp recordings. Signals were monitored on a digital oscilloscope, digitized (Digidata 1322A, Molecular Devices), and sampled at 20 KHz for storage on hard-disk using pClamp 8.2 software package (Molecular Devices). Current-clamp recordings were filtered at 10 kHz, and voltage-clamp data were filtered at 2 kHz. Continuous recordings were also stored for offline analysis onto VHS tape (Neurocorder DR-886, Cygnus Technologies, Delaware Water Gap, PA, USA).

Recorded cells were located in layer II near the border with layer I and recordings were accepted if the resting membrane potential was ≤−50 mV (cells with higher resting potentials tend not to be viable). None of the neurons sampled showed the burst-firing response to positive current injection that is characteristic of layer V PaS cells [23]. For current clamp experiments, series resistance was estimated by compensating for the discontinuity in the voltage response to −50 pA current pulses, and recordings were accepted if series resistance was <30 MΩ (mean: 13±1 MΩ). Changes in input resistance were monitored regularly using 500 ms hyperpolarizing current pulses (−100 pA, 0.1 Hz). For voltage clamp recordings, series resistance was estimated by cancellation of the fast component of whole-cell capacitive transients using a −2 mV voltage step, and was typically compensated ∼40–60% (range: 10–15 MΩ; mean: 14±1 MΩ). Series resistance was monitored throughout the experiment, and the recordings were discontinued if this value changed by ≥15%. The liquid junction potential was measured and found to be 7.6 mV [24], but the correction was not applied as incomplete dialysis within the extensive processes of parasubicular neurons may make the correction less accurate [25].

Electrophysiological characteristics of PaS neurons were analyzed using the Clampfit 8.2 software package (Clampfit 8.2, Molecular Devices). Spike properties were derived from the first action potential evoked in response to a minimal-amplitude 500 ms positive current injection, and action potential amplitude was calculated from resting membrane potential. Action potential width, and the amplitudes of fast and medium afterhyperpolarizations (fAHP and mAHP) were measured relative to action potential threshold (with a rate of >100 mV/ms) using previously established criteria [26], [27]. Input resistance was determined from the peak voltage response to a 500 ms, −100 pA current pulse from a holding level of −60 mV. Inward rectification was quantified as the ratio between peak input resistance determined from the peak voltage response to a 500 ms, −200 pA hyperpolarizing pulse and the steady-state input resistance determined from the voltage response at the end of the hyperpolarizing pulse [13]. Similarly, the anodal break potential was measured as the peak depolarization following the offset of a −200 pA pulse relative to baseline voltages. Spike frequency adaptation was assessed using 500-ms +100 pA current steps, and the adaptation index (AI) was computed according the formula: AI = 1−*f_f_*/*f_i_*, where *f_f_* is the final frequency, measured by final interspike interval (ISI) at end of intracellular current pulse, and *f_i_* is the initial frequency, measured using the ISI between first two action potentials [28].

Subthreshold membrane potential oscillations were assessed by depolarizing cells to near-threshold voltages using positive constant current injection for ≤30 s. Power spectra were computed using multitaper methods within the Chronux toolbox (http://chronux.org, [29]) and custom Matlab routines (Matlab 7.10, MathWorks, Natick, MA, USA). Samples were reduced to an effective sampling rate of 2 kHz and filtered (0.5–500 Hz), and the power spectrum for each cell was computed as the average squared magnitude of the fast Fourier transform across three 2.1-s non-overlapping recordings that contained no action potentials with a frequency resolution of 0.06 Hz. Membrane potential oscillations in PaS neurons at room temperature have a lower frequency than those at higher temperatures without alterations in power [11], and the power of oscillations was therefore calculated between 1.5–5.9 Hz and expressed as a percentage of the total power (0.1–500 Hz). Neurons were considered non-oscillatory if they did not show a doubling of power and clear peak between 1.5–5.9 Hz in the power spectrum when depolarized from rest to subthreshold voltages [11], [12].

The conductances underlying CCh-induced depolarization were assessed in voltage clamp experiments by use of slow voltage-ramps at 2 min intervals [19], [30]. The holding potential for voltage-clamp experiments was −60 mV. Voltage ramp protocols were preceded by a 1-s fixed step to −120 mV, followed by a 4-s linear depolarization to −40 mV. Currents elicited by CCh were computed by subtraction of ramp-evoked current traces during drug application from control traces.

Statistical analyses assessed alterations in electrophysiological properties after pharmacological manipulations using one-way repeated measures ANOVAs, paired t-tests, and significant effects were investigated using pairwise multiple comparisons using Student-Newman-Keuls method for parametric data, and Mann-Whitney *U* tests for nonparametric data between baseline and drug conditions, unless otherwise indicated. Data are presented as means±SEM.

### Immunohistochemistry

The staining of biocytin-filled neurons in intact slices has been reported previously in detail elsewhere [31]. Following completion of electrophysiological recordings, individual slices were fixed in 4% paraformaldehyde in 0.1 M sodium-phosphate buffer (NaPB, ph: 7.5) for ∼24 h at 4 °C, and stored in 0.1 M NaPB for 2–4 weeks. Slices were rinsed 3x (5 min per wash) in 0.1 M NaPB, and incubated in 1% H_2_O_2_ for 30 min to block endogenous peroxidases. To block non-specific binding, slices were then rinsed and incubated in PHT (0.1 M sodium phosphate buffer containing 1% heat-inactivated normal goat serum and 0.3% Triton X-100, pH: 7.5) for 2 h. The slices were subsequently transferred to an avidin-biotin-horseradish-peroxidase complex (ABC kit, Vector Labs, Burlington, ON, CA) in 0.1 M NaPB overnight at room temperature on an oscillating table. After six successive 1 h rinses in PHT, the slices were incubated in a Tris-buffered saline solution containing 0.01% H_2_O_2_, 0.5% 3,3′-diaminobenzidine (DAB), and 0.02% NiSO_4_ for 10–15 min. The staining reaction was quenched by rinsing the slices 3x (10 min per wash) in 0.1 M TBS. Slices were then dehydrated in progressive concentrations of glycerol (25%, 50%, 75%, 100%), and stored at 4 °C in 100% glycerol, and mounted on glass slides.

Biocytin-labeled neurons were reconstructed using the Neurolucida neuron tracing system (MBF Bioscience, Williston, VA, USA) with an upright microscope (Leica, DM-5000B, Concord, ON, CA) and digital camera (Hamamatsu ORCA-ER, Hamamatsu Photonics Deutschland, Bridgewater, NJ, USA). Photomicrographs were also obtained at multiple positions on the Z-axis (Leica, DFC-480) and two-dimensional composites were digitally-reconstructed using Adobe Photoshop CS3 software (Adobe Systems Inc., San Jose, CA, USA).

### Pharmacological manipulations

Drugs were stored in frozen stock solutions mixed in dH_2_O at 120 to 240 times the final concentrations, and diluted to final concentrations in ACSF prior to recordings. The effects of cholinergic receptor activation on layer II PaS neurons were assessed using bath application carbachol (carbamylcholine chloride; CCh, 5–50 µM) [14]. To determine whether cholinergic depolarization of PaS neurons was dependent on increases in local synaptic transmission, fast ionotropic synaptic transmission was blocked using 6-cyano-7-nitroquinoxaline-2,3-dione (CNQX, 20 µM), DL-(±)-2-amino-5-phosphonopentanoic acid (AP5, 50 µM), and bicuculline methiodide (10 µM). Cholinergic effects on muscarinic receptors were assessed using the nonselective antagonist atropine sulfate (1 µM), the M_1_-preferring blocker pirenzepine dihydrochloride (1 µM), and the M_2_ antagonist methoctramine tetrahydrochloride (1 µM). The contribution of Na^+^ conductances to cholinergic depolarization was assessed using the Na^+^ channel blocker tetrodotoxin (TTX, 0.5 µM). The muscarinic-sensitive KCNQ Kv7.2/3 channel *I*_M_ was blocked using 10,10-bis(4-pyridinylmethyl)-9(10H)-antracenone (XE-991, 10 µM). In voltage-clamp experiments, the potent *I*_h_ channel blocker 4-ethylphenylamino-1,2-dimethyl-6-methylaminopyrimidinium chloride (ZD7288, 50 µM) and TTX (0.5 µM) were routinely added to the bath. Additional potassium conductances were blocked with the wide-acting K^+^ blocker barium sulfate (200 µM). When barium was added to the bath, PO_4_ and SO_4_ were removed from ACSF. Most drugs were obtained from Sigma-Aldrich (St. Louis, MO, USA) except for pirenzepine, XE-991, ZD7288, and bicuculline methiodide, which were purchased from Ascent Scientific (Princeton, NJ, USA).

## Results

### Properties of morphologically identified stellate and pyramidal neurons

**Figure 1:**
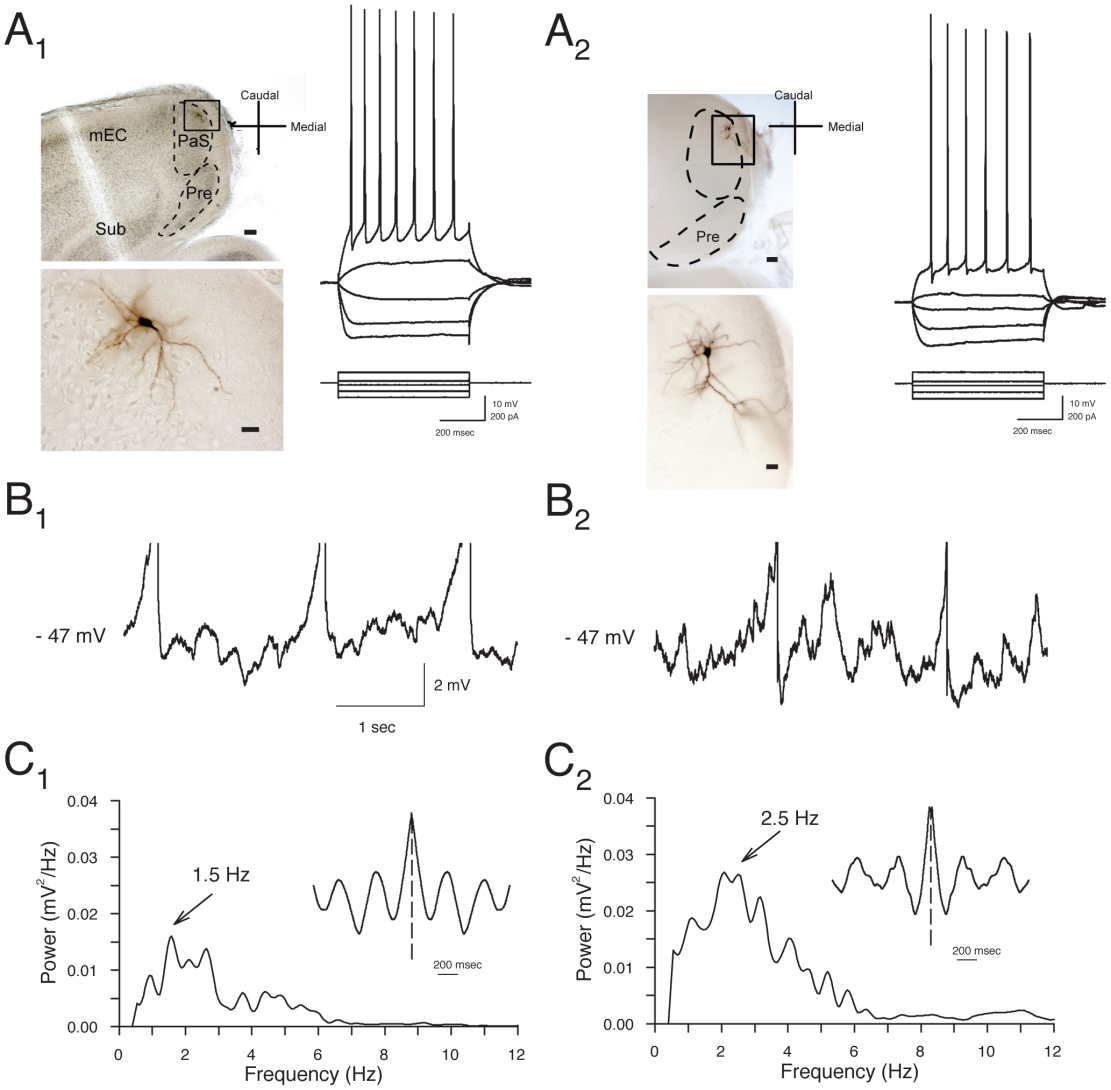
Layer II parasubucular (PaS) stellate and pyramidal neurons show similar electrophysiological properties. **A.** Photomicrographs of representative layer II stellate cell (A_1_) and pyramidal cells (A_2_) show cell bodies in layer II near the border with layer I. Calibration bars are 100 µm in top panels, and 20 µm in bottom panels. Membrane potential responses to hyperpolarizing and depolarizing current pulses for the cells shown were similar for stellate and pyramidal neurons (right panels). **B**. Both stellate (B_1_) and pyramidal (B_2_) neurons display theta-frequency membrane potential oscillations at near-threshold voltages. Representative traces obtained from the same cells as in A show oscillations in membrane potential when cells were depolarized to −47 mV using positive current injection. Note that action potentials are truncated. **C**. Power spectra reflect a peak frequency of oscillations at 1.5 to 5.9 Hz in these neurons recorded at 22–24 °C. The rhythmicity of oscillations is also reflected in the inset autocorrelograms.

Stable whole-cell recordings were obtained from 122 layer II PaS cells, and morphological identification of biocytin-filled neurons was obtained for 13 pyramidal neurons and 5 stellate neurons. Morphological characteristics of the layer II neurons were similar to those observed in previous extensive reports [22], [23], [32]. Stellate cells had 3 to 5 basal dendrites emanating from the soma that bifurcated several times in a pattern restricted to the superficial layers (Fig. 1A_1_). Stellate cells typically had spiny dendrites throughout all processes [23]. Pyramidal cells typically had multiple basal dendrites that occasionally extended to the border between layer IIIs and IV and one or two apical dendrites that extended to layer I (Fig. 1A_2_). In some cases, axons were traced out of the PaS via layer I, and could be observed in layer II of the entorhinal cortex, but no axons were observed descending to the deep layers. Pyramidal and stellate cells did not differ significantly in somatic diameter (16.6±1.4 µm for pyramidals vs. 14.0±1.3 µm for stellates; n.s., *p* = 0.19) or overall length of dendritic arbor (2046±409 µm for pyramidals vs. 2437±296 µm for stellates; n.s., *p* = 0.45).

The electrophysiological properties and firing patterns of the group of 122 layer II neurons were similar to those reported previously [11], [12], [23], and properties of identified pyramidal and stellate neurons were also quite similar. Layer II cells were typically quiescent at resting membrane potential (mean: −59.8±0.5 mV) and, in contrast to subicular and deep layer PaS neurons that can show burst firing [23], [32], both cell types typically responded to depolarizing current injection (+100 pA, 500 ms) with repetitive regular spiking and mild spike frequency adaptation (Fig. 1A, right panel). Morphologically-identified pyramidal and stellate neurons showed similar spike-frequency adaptation index values (see Methods) in response to +100 pA 500 ms depolarizing current steps (0.39±0.07 vs. 0.32±0.12, respectively; n.s., p = 0.63). Neurons exhibited high firing thresholds (mean: −39.5±0.4 mV), and action potentials (amplitude: 125.0±0.9 mV; duration: 3.5±0.1 ms) were typically followed by fast and medium latency afterhyperpolarizations (mean: 8.6±0.3 mV and 6.1±0.2, mV respectively). The mean input resistance of neurons (mean: 122.5±4.0 MΩ for the group of 122 cells) did not vary between morphologically identified pyramidal and stellate cells (108.0±4.3 MΩ and 109.7±7.1 MΩ, respectively; *p* = 0.84). There was moderate time-dependent inward rectification in response to hyperpolarizing current pulses in the group of 122 cells (sag ratio: 1.21±0.02), and the ratio did not vary between identified cell types (1.20±0.01 in pyramidal cells vs. 1.15±0.02 in stellate cells; n.s., *p* = 0.28). The offset of −200 pA pulses was typically followed by a depolarizing response (mean: 4.6±0.3 mV), and the amplitude of the anodal break potentials did not differ significantly between cell types. (4.0±0.4 mV in pyramidals vs. 3.4±0.2 mV in stellates; n.s., *p* = 0.37).

Many PaS neurons display intrinsic voltage-dependent theta-frequency oscillations in membrane potential [11], [12], and the majority of layer II PaS neurons tested in current clamp experiments (83.7%, 62 of 74 cells tested) also showed membrane potential oscillations at near-threshold voltages that accounted for 52.5±1.5% of total power (0.74±0.06 mV^2^/Hz between 1.5 and 5.9 Hz). Biocytin-filled stellate (Fig. 1B_1_) and pyramidal neurons (Fig. 1B_2_) both showed prominent oscillations but the pyramidal neurons displayed significantly higher levels of theta band power compared to stellate cells (1.32±0.25 in pyramidals vs. 0.42±0.07 mV^2^/Hz in stellates; *p*<0.05), and theta-band frequencies in pyramidal cells accounted for a larger portion of the total power compared to stellate cells (61.7±2.0% vs. 47.9±0.05%; *p*<0.01). Pyramidal neurons also showed slightly higher frequencies of membrane potential oscillations compared to stellate cells (2.9±0.3 Hz vs. 2.4±0.1 Hz; *p*<0.05).

### Cholinergic effects on resting potential and firing properties

**Figure 2:**
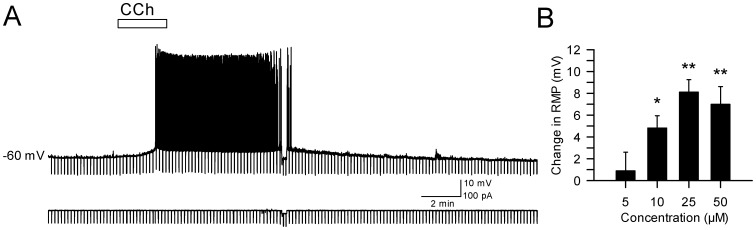
Carbachol depolarizes parasubicular neurons to threshold voltages. **A.** Representative recording of membrane potential from a layer II parasubicular neuron (same cell as in Figure 1A_1_) in response to a 2.5 min bath application of 25 µM CCh (white bar). Note that repeated hyperpolarizing current pulses were used to monitor input resistance during recordings, and that steady negative hyperpolarizing current was used to return the cell to baseline voltages to assess changes in input resistance. **B**. Group data show the mean depolarization of resting membrane potential in response to different concentrations of CCh in separate groups of neurons. The lowest concentration of 5 µM CCh failed to produce a significant depolarization, but higher doses reliably depolarized layer II PaS neurons (*: *p*<0.05; **: *p*<0.01).

The effects of cholinergic receptor activation on resting membrane potential was determined using bath application of carbachol (CCh, 5–50 µM) for 2–5 min (Fig. 2). Low doses of CCh (5 µM) added to normal ACSF did not result in significant changes in resting membrane potential (0.9±1.7 mV, *n* = 3), but higher doses of 10 to 50 µM CCh resulted in a slow depolarization in 62 of 77 cells (80.5% of cells). Mean depolarization increased with CCh concentration compared to the resting membrane potential in control ACSF (10 µM: 4.8±1.1 mV, N–K: *p*<0.05, *n* = 4; 25 µM: 8.1±1.1 mV, N–K: *p*<0.001, *n* = 14; 50 µM: 7.0±1.6 mV, N–K: *p*<0.001, *n* = 14; main effect of CCh: *p*<0.001; Fig. 2). The effects of CCh were maximal at 25–50 µM, and subsequent experiments therefore used these higher concentrations (see below). The majority of both pyramidal (9 out of 10 cells) and stellate (3 out of 5 cells) neurons responded to CCh with depolarization of resting membrane potential, however the mean level of depolarization did not differ between cell types (*p* = 0.94).

**Figure 3:**
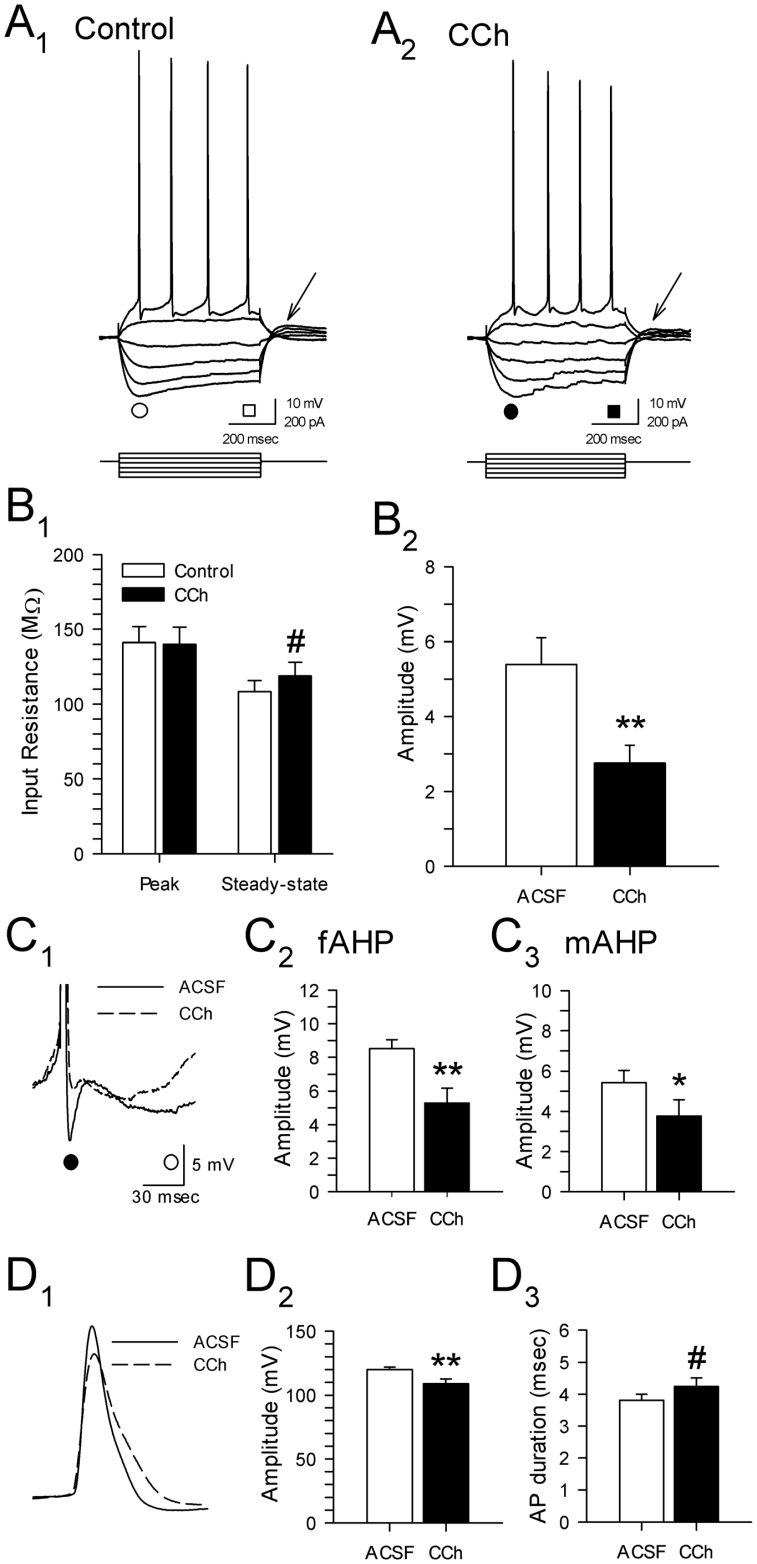
Carbachol has multiple effects on electrophysiological properties of layer II parasubicular neurons. **A.** Membrane voltage responses to hyperpolarizing and depolarizing current pulses in the same cell during perfusion with control ACSF (A_1_) and 25 µM CCh (A_2_). **B**. Group data in B_1_ show that CCh (25 µM) resulted in an increase in steady-state input resistance (measured at times indicated by squares in A; *: *p*<0.05), but had no effect on peak input resistance (see circles in A). Carbachol consistently reduced the amplitude of anodal break potentials following −200 pA steps (B_2_, **: *p*<0.01; see arrows in A). **C**. Magnified superimposed traces (C_1_) in control ACSF (solid line) and in the presence of 25 µM carbachol (dashed line) show a reduction in the average amplitude of the fast (C_2_; •, fAHP, **: *p*<0.01) and the medium afterhyperpolarization (C_3_; ○, mAHP, *: *p*<0.05). **D**. Superimposed action potentials show that carbachol (25 µM) was also associated with a significant reduction in action potential amplitude (C_1,2_; **: *p*<0.01) and a trend towards increased spike duration (C_3_; #: *p* = 0.06).

Changes in input resistance induced by CCh were investigated using −100 pA current pulses from a holding voltage level of −60 mV using steady-state hyperpolarizing current. Application of CCh (25 µM) caused no appreciable change in peak input resistance (145.5±11.2 vs. 138.2±11.2 MΩ; *p* = 0.63), but was associated with a significant increase in steady-state input resistance (123.7±8.7 vs. 108.5±7.3 MΩ; *p*<0.05; Fig. 3A,B_1_). The slight increase in steady-state vs. peak input resistance observed here is consistent with inhibition of non-ohmic K^+^ currents such as *I*_Kir_ [18].

Carbachol induced a dose-dependent reduction in hyperpolarization-induced inward rectification, consistent with recent work suggesting a cholinergic modulation of theta frequency resonance via a reduction in *I*_h_ [33], [34]. There was no significant effect of 25 µM CCh on sag ratio (1.24±0.07 in CCh vs. 1.26±0.05 in ACSF; *n* = 14; *p* = 0.41), but 50 µM CCh induced a small reduction in the ratio (1.10±0.04 vs. 1.17±0.03; *p*<0.05, *n* = 7; data not shown).

Anodal break potentials were reduced by both 25 µM CCh (2.7±0.5 vs. 5.4±0.7 mV; *p*<0.01; Fig. 3B_2_) and 50 µM CCh (1.6±0.4 vs. 3.8±0.7 mV; *p*<0.05). Both *I*_h_ and a voltage-dependent Na^+^ conductance [35] can contribute to anodal break potentials, and the contribution of Na^+^ currents to the CCh-induced reduction in the potentials was therefore assessed in tests using the Na^+^ channel blocker TTX (0.5 µM). Application of TTX greatly reduced the amplitude of the anodal break potentials from 4.8±1.2 mV to 0.7±0.4 mV, while subsequent application of CCh did not lead to any further reductions (*n* = 3, data not shown), suggesting that CCh-induced reductions anodal break potentials are likely due to an attenuation of voltage-dependent Na^+^ conductances, rather than a suppression of *I*_h_.

Carbachol also had strong effects on the firing of layer II PaS neurons. Carbachol (25 µM) reduced the amplitude of both fast (4.9±0.9 vs. 8.9±0.6 mV; *p*<0.01) and medium afterhyperpolarizations (2.9±0.7 vs. 5.2±0.6 mV; *p*<0.01; Fig. 3C), and also led to a significant reduction in the spike amplitude (107.9±4.3 vs. 122.3±2.0 mV; *p*<0.01; Fig. 3D_1_). The reduction of spike amplitude and the suppression of the fAHP was associated with a nonsignificant increase in action potential duration (4.1±0.3 vs. 3.7±0.2 ms; *p* = 0.06, Fig. 3D_2_). These effects were reversed by a 15 min washout period in a subset of 6 cells tested (data not shown).

### Cholinergic depolarization is not mediated by changes in synaptic inputs

Cholinergic receptor activation depolarizes neurons that project to the PaS and the contribution of changes in synaptic input to the cholinergic depolarization of PaS cells was assessed using coapplication of CCh with CNQX (20 µM), AP-5 (50 µM), and bicuculline methiodide (25 µM) to block AMPA and kainate, NMDA, and GABA_A_-mediated synaptic transmission, respectively. Blockade of fast glutamatergic and GABAergic receptors failed to block the CCh-induced depolarization, and bath application of CCh (50 µM) in the presence of the synaptic blockers depolarized neurons from −56.2±0.7 to −48.4±1.8 mV (*n* = 5, *p*<0.01; data not shown). Membrane potential returned to baseline voltages (mean: −57.2±1.1 mV) after drug washout. Alterations in fast excitatory and inhibitory transmission are therefore not required for the CCh-induced depolarization of layer II PaS neurons, suggesting that CCh directly affects intrinsic conductances in PaS neurons.

### CCh-induced depolarization is dependent on muscarinic receptors

**Figure 4:**
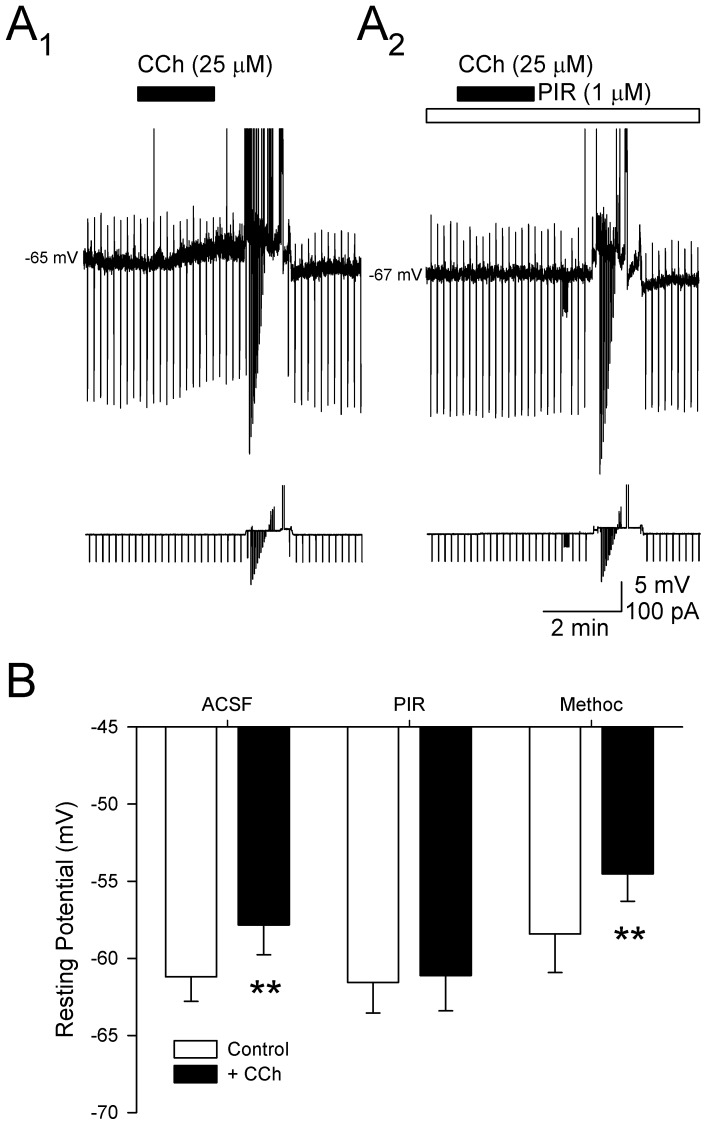
The depolarization of layer II parasubicular neurons induced by carbachol (CCh) is likely mediated by M_1_ receptors. **A.** Voltage traces from a representative PaS neuron show that application of CCh alone (50 µM; black bar) depolarizes membrane potential (A_1_), and that 15-min bath application of the selective M_1_ receptor antagonist, pirenzepine (PIR; 1 µM; white bar) blocked the depolarization induced by CCh. Note that hyperpolarizing and depolarizing current pulses were used to assess alterations in input resistances and cell firing from a potential of −60 mV. **B**. Group data show that the depolarization of membrane potential induced by application of 50 µM CCh was blocked by pirenzepine (PIR; 1 µM), but not by the M_2_ receptor blocker, methoctramine (Methoc; 1 µM), suggesting that M_1_, but not M_2_ receptors, mediate the cholinergic depolarization of layer II PaS neurons (**: *p*<0.01).

Initial tests showed that addition of the muscarinic receptor antagonist atropine (1 µM) to the bath for 15–20 min prior to CCh (50 µM) completely blocked the CCh-induced depolarization (-58.2±1.9 mV in atropine vs. −57.7±1.8 mV with addition of CCh; *n* = 7, *p* = 0.67), indicating that muscarinic receptors are required (data not shown). Co-application of more specific antagonists was then used to assess the muscarinic receptors involved. High concentrations of CCh (25–50 µM) were first used to verify that cells showed a reversible depolarization in response to CCh alone (−61.4±1.5 mV in ACSF vs. −56.2±1.8 mV in CCh; *n* = 6, N–K: *p*<0.01, Fig. 4A_1_). Subsequent application of the M_1_–preferring antagonist pirenzepine (1 µM) for a period of 15 min completely blocked CCh-induced membrane depolarization (−60.1±2.0 mV in pirenzepine vs. −60.2±1.8 mV with CCh; N–K: *p* = 0.73; Fig. 4A_2_). The M_2_-preferring antagonist methoctramine (1 µM), however, failed to block the CCh-induced depolarization (−58.4±2.5 mV in methoctramine vs. −54.5±1.7 mV with CCh; *n* = 3, N–K: *p*<0.05; Fig. 4B) suggesting that the CCh-induced depolarization is dependent primarily on M_1_-like receptors. Further, the effects of CCh on action potential amplitude, afterhyperpolarizations and width were also blocked by application of atropine (1 µM, *n* = 7; AP amplitude: 122.4±4.2 vs. 125.5±4.0 mV, *p* = 0.26; AP width: 3.7±0.3 vs. 3.5±0.4 ms, *p* = 0.1; fAHP: 6.3±1.8 vs. 8.2±1.9 mV, *p* = 0.11) and pirenzipine (1 µM; *n* = 6; AP amplitude: 125.5±2.2 vs. 127.8±0.8 mV, *p* = 0.16; AP width: 3.2±0.5 vs. 3.1±0.4 ms, *p* = 0.17; fAHP: 3.4±1.4 vs. 3.3 mV±0.5, *p* = 0.13), suggesting that these changes are dependent on M_1_ receptors.

### Cholinergic depolarization is mediated by I_M_ and an additional K^+^ conductance

**Figure 5:**
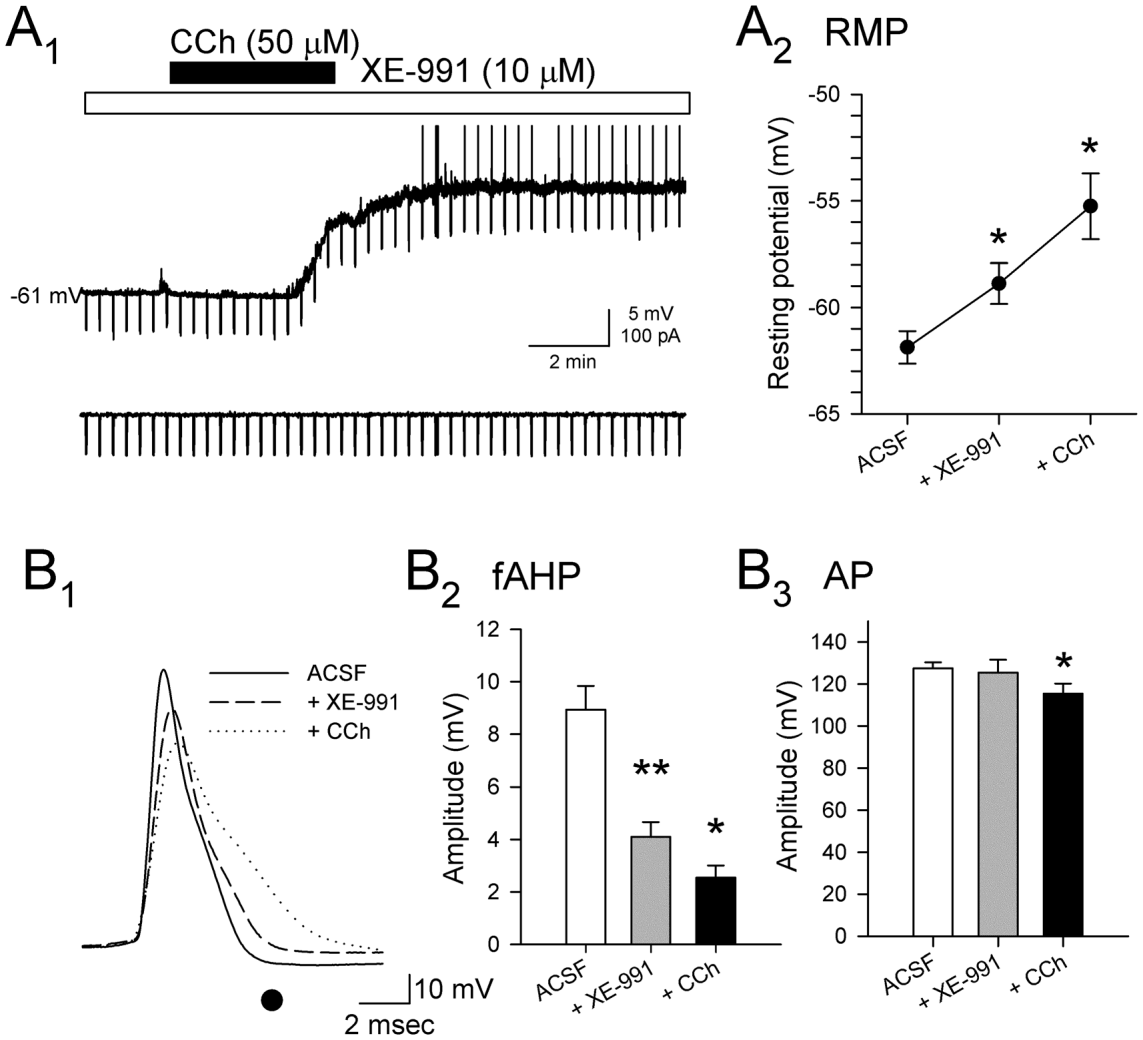
Carbachol-induced depolarization and alterations in spike properties are not mediated solely by inhibition of *I*_M_. **A.** An example recording of membrane potential in the presence of synaptic antagonists and the selective Kv7.2/3 antagonist XE-991 (10 µM) shows a depolarization in response to bath application of CCh (A_1_; white bar; 50 µM) resulted in additional depolarization, suggesting that an additional current also contributes to the CCh-induced depolarization. Group data also reflect the significant depolarization induced by CCh in the presence of XE-991 (10 µM) and synaptic antagonists (A_2_; *: *p*<0.05; **: *p*<0.01). **B**. Comparison of action potentials (B_1_) recorded in XE-991 alone (dashed line) and following addition of CCh (dotted line) suggests that changes in spike morphology are mediated in part by conductances that are not dependent on *I*_M_. Addition of CCh results in additional reductions in the amplitude of the fAHP (measured at • in B_1_) compared to XE-991 alone (B_2_; *: *p*<0.05; **: *p*<0.01). Action potential amplitude (B_3_) is not significantly affected by XE-991 alone (grey bar), but is reduced when CCh was added (black bar; *: *p*<0.05).

Inhibition of the voltage-dependent Kv7.2/3-mediated current, *I*_M_, as well as inhibition of a voltage-independent K^+^ leak current contributes to the cholinergic depolarization of hippocampal neurons [18], and previous studies using in situ hybridization have demonstrated that the PaS expresses moderately high levels of KCNQ2/3 channels [36]. To determine whether the depolarization of PaS neurons was dependent on inhibition of *I*_M_, the selective Kv7.2/3 channel blocker XE-991 (10 µM) was bath applied in the presence of synaptic antagonists for 10–15 min prior to application of CCh (25–50 µM). Bath application of XE-991 depolarized layer II PaS neurons by 3.0±0.6 mV (N–K: *p*<0.01), indicating that PaS neurons are sensitive to blockade of *I*_M_. Subsequent application of CCh resulted in a further depolarization of 3.6±0.8 mV (N–K: *p*<0.01, *n* = 5), suggesting that a blockade of *I*_M_ cannot fully account for CCh-induced depolarization of membrane potential (Fig. 5A).

Blockade of *I*_M_ with XE-991 also led to decreases in the amplitude of fast- and medium duration AHPs (fAHP, N–K: *p*<0.01; mAHP, N–K: *p*<0.05; Fig. 5B_1,2_) [27], [37]. Spike amplitude was not reliably affected by XE-991 alone, but the addition of CCh resulted in a significant reduction of spike amplitude from 125.5±6.1 mV to 115.4±4.9 mV (N–K: *p*<0.05; Fig. 5B_3_), suggesting that effects of CCh on conductances that mediate action potentials are not exclusively due to alterations of *I*_M_.

**Figure 6:**
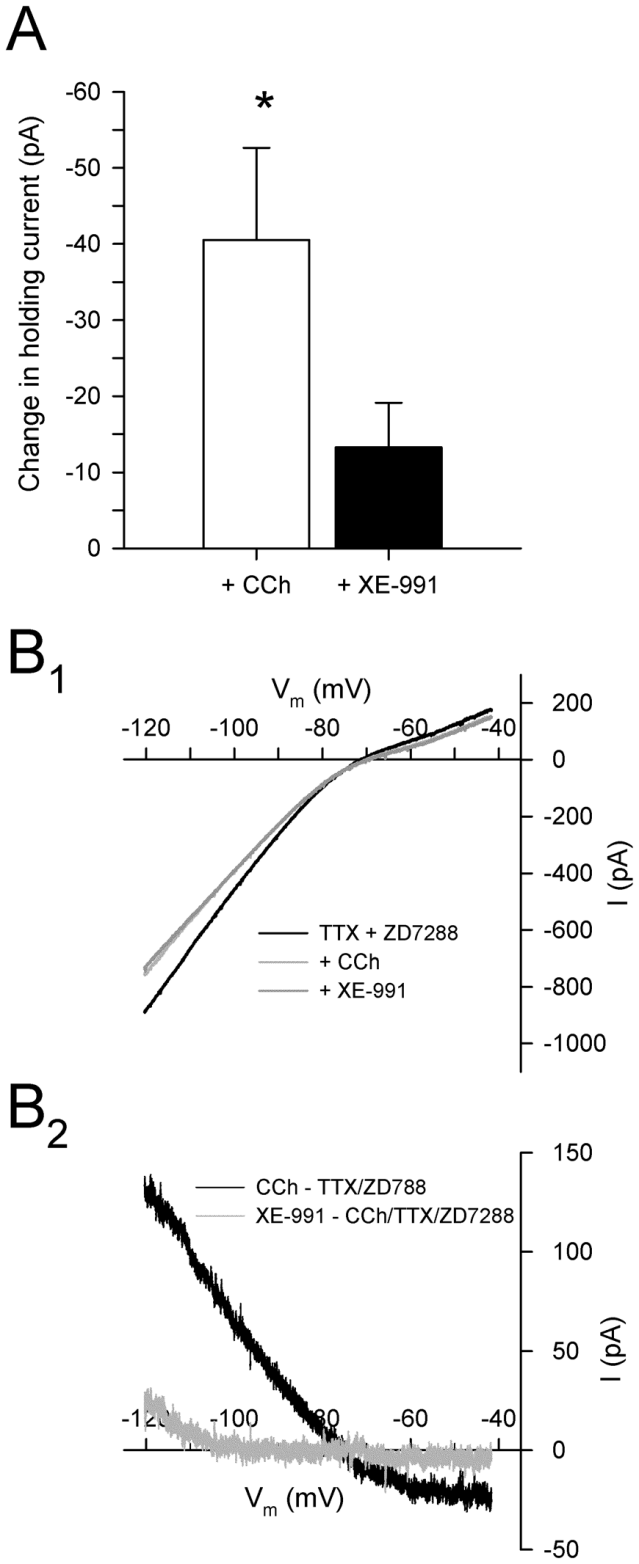
Cholinergic depolarization of PaS neurons is dependent in part on blockade of the muscarinic-dependent outward K^+^ current, *I*_M_. **A.** Bath application of CCh (50 µM) results in a significant inward current in cells held at −60 mV (*: *p*<0.05), and also occludes inward currents induced by the M-current blocker XE-991 (10 µM; black bar, compare with Fig. 5A). **B.** Membrane currents during slow voltage ramps from −120 mV to −40 mV in the presence of TTX (0.5 µM) and ZD7288 (50 µM), and during subsequent bath application of CCh (light grey line; 50 µM; B_1_) show that cholinergic receptor activation induces an inward current at voltages near resting membrane potential that reverses around −76 mV, consistent the blockade of outward K^+^ currents. The application of CCh occluded additional inward current during subsequent bath application of XE-991 (dark grey line; 10 µM) suggesting that CCh depolarizes PaS neurons in part by suppression of the M-current. Current subtractions show that CCh blocks an outward current that reverses at −83.3±7.0 mV (black line; B_2_), and also occluded membrane currents normally induced by XE-991 (grey line).

The ionic conductances that are modulated by cholinergic receptor activation were assessed in voltage clamp experiments in the presence of TTX (0.5 µM) and ZD7288 (50 µM) to block voltage-dependent activation of sodium channels and the hyperpolarization-activated current *I*_h_, respectively. Application of ZD7288 and TTX (*n* = 14) increased steady-state input resistance from 87.0±7.9 to 103.1±11.3 MΩ (*p*<0.05) without affecting peak resistances (103.7±11.3 vs. 99.9±9.6 MΩ; *p* = 0.50), and completely abolished inward rectification (sag ratio: 1.00±0.00 vs. 1.18±0.05; *p*<0.05), however failed to induce any significant change in holding current at −60 mV (<5 pA). Consistent with the depolarization observed in current clamp experiments (Fig. 2), subsequent bath application of CCh (50 µM) resulted in a large inward current in cells held at −60 mV (−40.5±12.1 pA; *n* = 5; *p*<0.05; Fig. 6A); the increase in mean input resistance from 107.9±23.5 to 119.0±25.9 MΩ, however, was not statistically significant (*p* = 0.12).

The currents responsible for the cholinergic depolarization were characterized using slow voltage ramps from −120 mV to −40 mV (20 mV/s) in the presence of synaptic blockers and by subtracting current responses to voltage ramps before and after drug application (Fig. 6B). The inward current induced by CCh reversed at −83.3±7.0 mV to an outward current at more negative voltages. These findings suggest that CCh depolarizes layer II PaS neurons through the blockade of a potassium conductance.

Results of current clamp experiments had shown that blockade of the M-current with XE-991 depolarized layer II parasubicular neurons (Fig. 5), suggesting that CCh-induced depolarization is mediated in part by attenuation of *I*_M_. If CCh attenuates *I*_M_, then the application of CCh should result in an occlusion of any further current induced by application of the *I*_M_ blocker XE-991. Blockade of *I*_M_ using XE-991 (10 µM) in the presence of CCh induced only a small inward current (−13.3±5.8 pA at −60 mV; *n* = 5; N–K: *p* = 0.32; Fig. 6), suggesting that the CCh-induced depolarization is mediated in part via blockade of *I*_M_.

**Figure 7:**
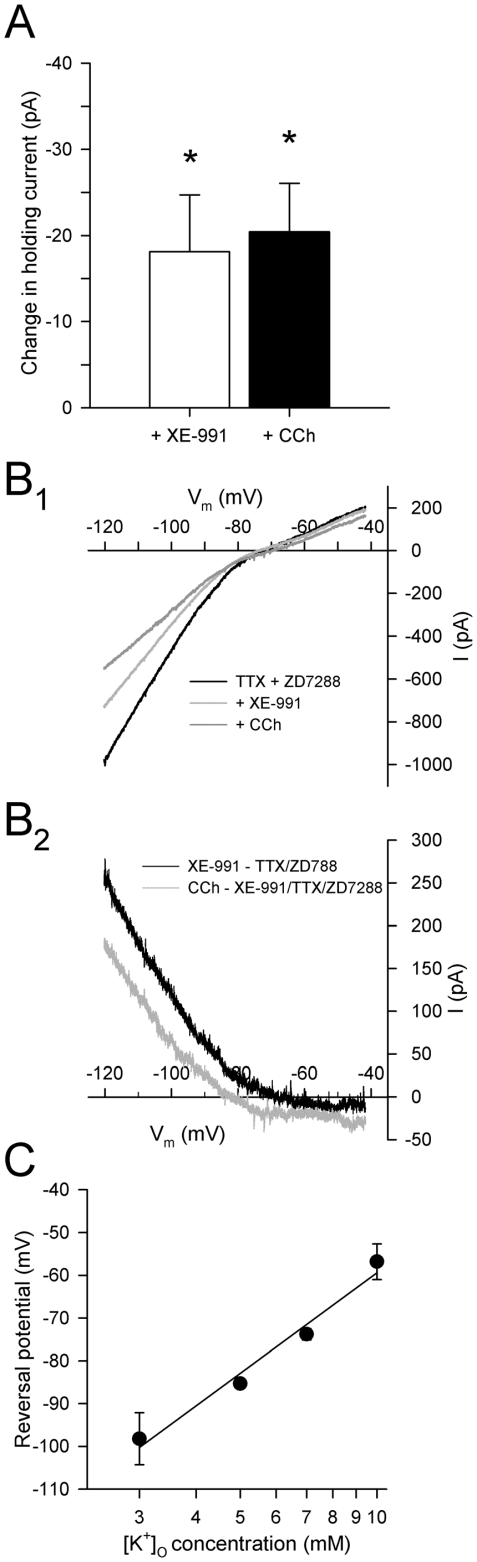
Cholinergic receptor activation induces a non-*I*_M_ inward current that is likely mediated by an attenuation of an inwardly rectifying K^+^ conductance. **A.** Bath application of XE-991 (10 µM; white bar) resulted in an inward current in cells held at −60 mV indicating that the cells express *I*_M_, and the subsequent perfusion with CCh (50 µM) resulted in an additional inward current, suggesting that CCh blocks a second K^+^ conductance (*: *p*<0.05). **B.** Current traces are shown for a PaS neuron in response to slow voltage ramps from −120 mV to −40 mV during bath application of TTX (0.5 µM) and ZD7288 (50 µM), subsequent application of XE-991 (light grey line; 10 µM), and subsequent application of CCh (dark grey line; 50 µM; B_1_). Current subtractions (B_2_) show that XE-991 induced an inward current at voltages near rest that reversed at −78.0±3.2 mV, and that application of CCh (50 µM) resulted in the blockade of an additional inward current that reversed at −85.3±1.1 mV. The estimated equilibrium potential for K^+^ was −84 mV, suggesting that the inward currents are mediated by an attenuation of K^+^ conductances. C. The reversal potential of the current induced by CCh in the presence of XE-991 (10 µM), TTX (0.5 µM), and ZD7288 (50 µM) is shifted in relation to extracellular K^+^ concentration. Substitution of 3 mM for 5 mM [K^+^]_O_ shifted the mean reversal potential to a more negative voltage, and 7 mM and 10 mM [K^+^]_O_ shifted the reversal potential to more positive voltages. The reversal potentials showed a linear relationship with the logarithmic [K^+^]_O_ concentration, suggesting that CCh induces an inward current through inhibition of a K^+^ current.

To assess the effects of blockade of *I*_M_ under voltage clamp conditions, and to determine if CCh acts through the blockade of an additional conductance other than *I*_M_, voltage ramps were performed in ACSF containing TTX (0.5 µM), ZD7288 (50 µM), prior to and during sequential addition of XE-991 (10 µM) and CCh (50 µM) (*n* = 9). Application of XE-991 in the presence of TTX and ZD7288 resulted in a significant increase in current required to hold neurons at −60 mV (−18.1±6.6 pA; N–K: *p*<0.05; Fig. 7A). The inward current reversed to an outward current at −78.0±3.2 mV, consistent with the effects of XE-991 being mediated in large part by a block of the outward K^+^ conductance *I*_M_ (Fig. 7B). Subsequent application of CCh in the presence of XE-991 resulted in an additional inward current at a holding potential of −60 mV (−20.4±5.6 pA; N–K: *p*<0.05). The current reversed at −85.3±1.1 mV and showed rectification at voltages positive to E_K_, suggesting that the inward current is due to attenuation of an inward rectifying K^+^ conductance (Fig. 7B). Taken together, these data indicate that CCh depolarizes PaS neurons through effects on *I*_M_ as well as an additional K^+^ conductance, likely mediated by inward rectifying K^+^ channels.

The inward current induced by CCh in the presence of XE-991 (see Fig. 7) reversed near the equilibrium potential for the K^+^ in normal ACSF with 5 mM K^+^ (−85.3±1.1 mV) suggesting that cholinergic receptor activation may help depolarize layer II parasubicular neurons via blockade of a potassium conductance. The dependence of this non-*I*_M_ current on K^+^ was further investigated by systematically varying the extracellular concentration of K^+^ in the presence of TTX (0.5 µM), ZD7288 (50 µM), and XE-991 (10 µM) while maintaining osmolarity of ACSF by adding or subtracting equimolar NaCl to compensate for changes in the amount of KCl used. When [K^+^]_O_ was reduced to 3 mM, the reversal potential of the CCh-induced current was shifted to −98.2±6.1 mV (*n* = 4), and raising the [K^+^]_O_ concentration to 7 mM (*n* = 10) and 10 mM (*n* = 6) shifted the reversal potential to −73.8±1.3 mV and −56.8±4.2 mV, respectively. The reversal potentials of the CCh-induced currents in layer II PaS neurons show a linear fit with the logarithmically plotted [K^+^]_O_ concentrations, indicating that the current induced by CCh in the presence of the *I*_M_ blocker XE-991 is due to inhibition of a potassium conductance [30] (Fig. 7C).

### Carbachol attenuates a Ba^2+^-sensitive K^+^ conductance

**Figure 8:**
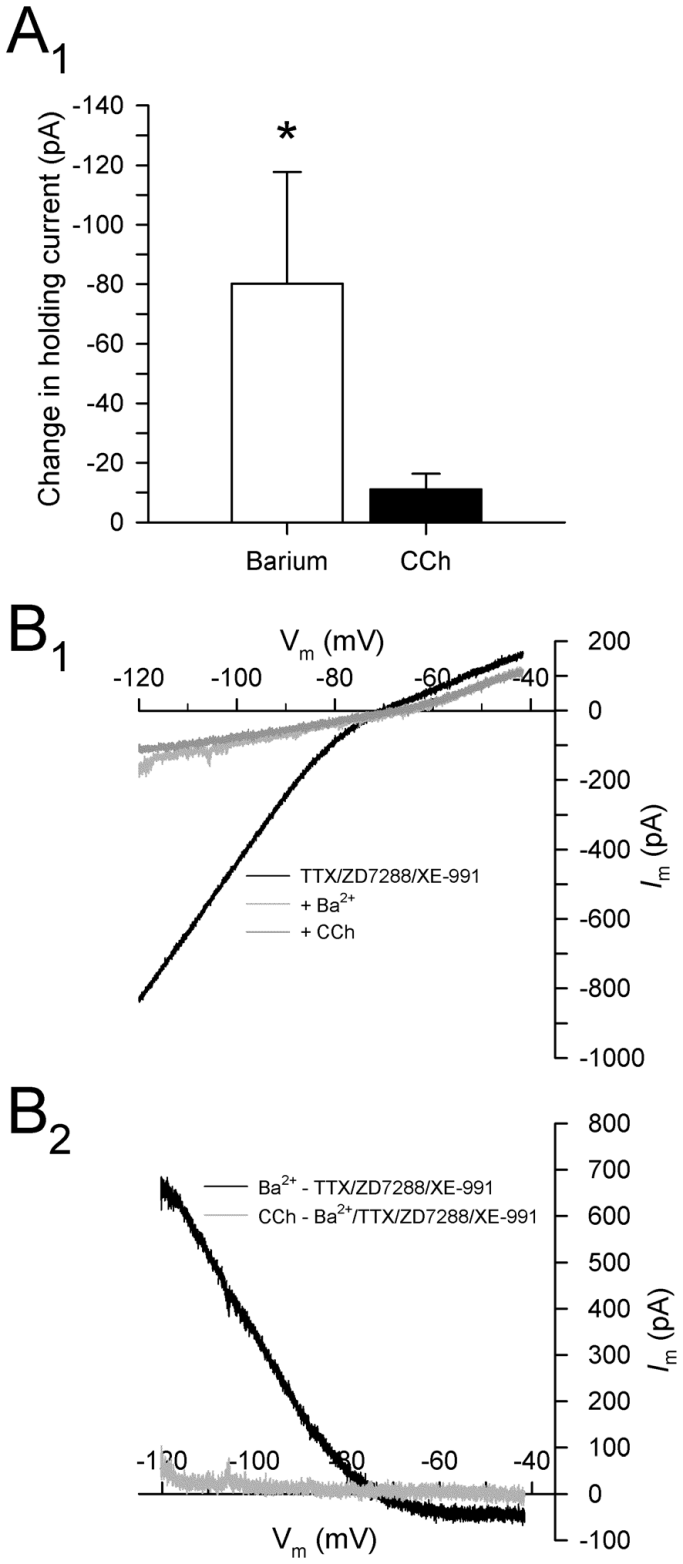
Cholinergic depolarization in layer II parasubicular neurons is mediated by an inhibition of a Ba^2+^-sensitive K^+^ conductance. **A.** Mean group data show that Ba^2+^ (200 µM) induces a strong inward current in cells held at −60 mV, and that CCh (50 µM) fails to induce any additional inward current in the presence Ba^2+^ (*: *p*<0.05). **B.** Current traces in response to slow voltage ramps from −120 mV to −40 mV are shown for tests in the presence of TTX (0.5 µM) and ZD7288 (50 µM), after the addition of Ba^2+^ (200 µM), and after the addition of CCh (50 µM) (B_1_). Current subtractions (B_2_) show that Ba^2+^ induced an inward current consistent with suppression of an outward K^+^ current at voltages negative to E_K_, and that subsequent application of CCh failed to elicit an additional current. The inward current induced by CCh in the presence of XE-991 (see Fig. 7) is therefore mediated by a Ba^2+^-sensitive K^+^ current.

To determine if the depolarizing current induced by CCh in the presence of XE-991 is dependent on K^+^, the effects of CCh were tested in the presence of XE-991 and Ba^2+^. Barium is a wide-acting K^+^ channel blocker that attenuates the voltage-independent *I*_leak_ and the voltage-sensitive *I*_Kir_, and also attenuates cholinergic depolarization in hippocampal neurons [17]. In the presence of TTX (0.5 µM), ZD7288 (50 µM), and XE-991 (10 µM), the addition of Ba^2+^ (200 µM) induced a large inward current at a holding potential of −60 mV (−80.1±37.6 pA; N–K: *p*<0.05; *n* = 5; Fig. 8A). Current subtractions of voltage ramps showed that the Ba^2+^-dependent current reversed at −80.2±1.7 mV, consistent with a block of K^+^ conductances. Subsequent application of CCh (50 µM) failed to induce any significant additional current (−11.1±5.3 pA at −60 mV; n.s., *p* = 0.72), suggesting that CCh depolarizes PaS neurons via attenuation a Ba^2+^-sensitive K^+^ current in addition to *I*_M_ (Fig. 8).

## Discussion

The results presented here show that cholinergic receptor activation has a strong depolarizing effect on resting membrane potential in layer II PaS neurons, and this contrasts sharply with the powerful muscarinic suppression of excitatory synaptic transmission that we have observed previously [38]. Bath application of CCh resulted in the slow depolarization of about ∼80% of morphologically-identified parasubicular stellate and pyramidal neurons. The depolarization was found to be due to activation of M_1_ muscarinic receptors, and comparison of currents induced during slowly ramped voltage commands indicate that the muscarinic depolarization is mediated via inhibition of *I*_M_ as well as an additional K^+^ current. The additional K^+^ current displayed an inwardly rectifying voltage-current profile similar to currents carried through Kir2.3 channels following muscarinic receptor stimulation in pyramidal neurons in the prefrontal cortex as well as in striatal neurons [39], [40], [41]. This contrasts with the mechanisms of cholinergic depolarization in other cortical pyramidal neurons and in layer II cells of the medial entorhinal cortex, which is due in part to activation of the Ca^2+^-modulated nonspecific cationic conductance *I*_NCM_ [15], [19].

### Cholinergic depolarization via muscarinic receptors

Although carbachol is known to exert strong actions on nicotinic cholinergic receptors in the parahippocampal region including the PaS [42], it is unlikely to contribute to the slow and sustained depolarization in membrane potential observed, which shows a time-course that is more consistent with muscarinic receptor activation. Further, this effect was completely blocked by low doses of the potent muscarinic antagonist atropine sulfate (1 µM), indicating that muscarinic receptors are necessary for sustained depolarization in layer II parasubicular neurons.

Autoradiographic studies in rats and nonhuman primates have demonstrated moderate binding densities of both M_1_ and M_2_ receptors in the superficial layers of the PaS [43]. The cholinergic depolarization observed here was blocked by the M_1_-preferring antagonist pirenzepine, but not by the M_2_–preferring antagonist methoctramine (Fig. 4). Pirenzepine binds to M_1_ receptors with much greater affinity than M_2−5_ receptors, and the concentration used here is near the minimal effective concentration reported for layer II entorhinal neurons (∼0.8 µM; [35]), but pirezepine also has a moderate affinity for M_4_ receptors [44], [45]. Carbachol-induced membrane depolarization was also observed in the presence of the M_2_-preferring antagonist methoctramine that also blocks M_4_ receptors [44], however, suggesting that M_4_ receptors are not necessary for the muscarinic depolarization of layer II PaS neurons. Thus, although both pirezepine and methoctramine have affinities for different muscarinic subtypes [45], the present data support the tentative conclusion that the CCh-induced depolarization is mediated primarily by actions on M_1_ receptors.

### Cholinergic modulation of action potentials and passive membrane properties

Consistent with previous findings, the electrophysiological properties of stellate and pyramidal PaS neurons were comparable [22], [23], [32], and they also responded similarly to CCh application. Both cell types reliably displayed rhythmic oscillations, and in contrast to layer II neurons of the adjacent entorhinal cortex, the amplitude of these oscillations was slightly larger in neurons with pyramidal-like morphology compared to those with stellate-like morphology [35], [46], [47]. Overall, these findings indicate a relative electrophysiological homogeneity amongst layer II parasubicular neurons [23].

Cholinergic receptor activation in the entorhinal cortex and the hippocampus is associated with pronounced alterations of action potential shape and duration [35], [48] and similar effects were observed here in layer II PaS neurons. Carbachol led to a significant reduction in the amplitude of both fast and medium AHPs, and was accompanied by a non-significant increase in action potential duration (Fig. 3) [27]. Muscarinic receptor activation can suppress conductances associated with action potential generation and repolarization, including spike-dependent Ca^2+^ influx and K^+^ conductances such as *I*_M_ and *I*_K_. Blockade of *I*_M_ with XE-991 reduces the amplitude of fast and medium AHPs in both entorhinal and PaS neurons [12], [37], and this indicates that CCh may suppress AHPs via inhibition of *I*_M_ (Fig. 5B).

Inhibition of spike-evoked Ca^2+^ currents by CCh may also have contributed to the spike broadening and reduction in fast AHPs observed here [35], [49], [50], [51], [52]. We have also observed previously in PaS neurons that fast AHPs are reduced in Ca^2+^-free ACSF or during Ca^2+^ channel blockade [12]. Alternately, carbachol may modulate Ca^2+^-dependent K^+^ channels, or inhibition of Ca^2+^ influx may suppress fAHPs via an attenuation of fast Ca^2+^-dependent K^+^ currents [48].

Bath application of CCh consistently reduced the amplitude of action potentials. In contrast, bath application of XE-991 did not cause a similar reduction, indicating that the reduction in spike amplitude is not mediated inhibition of *I*_M_. It is possible that CCh reduced action potential amplitude by reducing the number of available voltage-gated Na^+^ channels via increases in intracellular Ca^2+^ [35], [48], [53], [54].

High concentrations of CCh (50 µM) led to a small attenuation of inward rectification in PaS neurons, suggesting that CCh reduced *I*_h_. Muscarinic receptor activation has effects on *I*_h_ in layer II entorhinal stellate neurons, including suppression of *I*_h_-mediated tail currents, a negative shift in activation range, and effects on the frequency and strength of membrane potential resonance [33], [34]. Muscarinic M_1_ receptor activation can modulate the hyperpolarization-activated cyclic nucleotide-regulated (HCN) channels that carry *I*_h_ through activation of PLCß [55], and it is possible that a PLCß-mediated mechanism could contribute to the modest reduction in the sag responses observed here.

#### Conductances mediating cholinergic depolarization of layer II PaS neurons

Acetylcholine modulates a variety of K^+^ conductances, and M_1_ receptor stimulation is known to suppress several K^+^ conductances including *I*_M_, *I*_leak_, *I*_AHP_, *I*_K(Ca)_ and *I*_Kir_, and can also lead to activation of a Ca^2+^-modulated nonspecific cationic current [17], [18], [19], [39], [40]. Here, we present evidence that muscarinic depolarization in PaS neurons is dependent primarily on the inhibition of two K^+^ conductances: the voltage-dependent K^+^ current *I*_M_, and an inward rectifying K^+^ current.

The M-current is a low threshold, non-inactivating, voltage-gated K^+^ conductance composed of Kv7 (KCNQ; primarily Kv7.2/3) that is active at threshold potentials, and the inhibition of *I*_M_ is a powerful mechanism that modulates neuronal excitability by depolarizing membrane potential in numerous cell types [56], [57]. M_1_-receptor-mediated activation of PLC depletes membrane-bound PIP_2_, which can modulate the open probability of KCNQ channels that mediate *I*_M_ [58], [59], [60], [61]. Previous studies using in situ hybridization have demonstrated that the PaS expresses moderately high levels of KCNQ2/3 channels [36], and the application of the M-current blocker XE-991 depolarized the membrane potential of layer II PaS neurons through occlusion of an outward current (Figs. 5 and 6), strongly suggesting that these neurons express functional KCNQ channels that are open at resting potential [57]. Further, occlusion of *I*_M_ reduced the magnitude of the isolated CCh-induced current, suggesting that the depolarization induced by CCh is mediated, at least in part, by inhibition of *I*_M_ (Fig. 6). Although XE-991 depolarized parasubicular neurons, and also resulted in a net inward current in cells held in voltage clamp that reversed near E_K_, the unambiguous presence of *I*_M_ in parasubicular neurons will need to be directly tested in future experiments using previously established deactivation protocols, as well as through the use of specific M-current activators such as retigabine [34], [62]. In addition, there remains a possibility that some part of the effects observed here might be due to modulation of presynaptic transmitter release by XE-991 [63]. Further, XE-991 can reversibly block ERG1-2 K^+^ channels with an EC_50_ of 107 µM, but the 10 µM concentration used here is likely to have minimal effects on these channels [64].

Carbachol induced an additional depolarization when it was applied *following* application of XE-991 to block *I*_M_, indicating that the CCh-induced depolarization is mediated both by *I*_M_ as well as an additional conductance. It is unlikely that this result is due to an incomplete block of *I*_M_ by XE-991, because XE-991 evokes a maximal effect on KCNQ channels at submicromolar concentrations (∼0.5 µM) in hippocampal slices, and the much higher concentration used here (10 µM) should result in a complete block [65]. Subtraction of currents associated with slowly ramped voltage commands were used to identify the additional current in response to CCh during blockade of *I*_M_. The CCh-induced current in the presence of XE-991 reversed at −85.3±1.1 mV in ACSF containing 5 mM K^+^ (near the predicted equilibrium potential for K^+^ of −84.7 mV), and the reversal potential also shifted consistently with predicted equilibrium potentials as the extracellular K^+^ concentration was varied from 3 to 10 mM (Fig. 7C), indicating that the current is attributable to a K^+^ conductance.

The second K^+^ conductance is likely to be mediated by inward rectifying K^+^ channels (Kir2), which are open at resting potentials and contribute to the regulation of resting membrane potential. The current-voltage relationship of the CCh-induced current in the presence of XE-991 shows moderate rectification at potentials above E_K_, similar to previously characterized *I*_Kir_ currents [66], and in situ hybridization has shown that the PaS contains moderate levels of IRK3 mRNA that expresses the Kir2.3 channel protein [67]. Further, although Ba^2+^ can affect multiple K^+^ conductances, application of Ba^2+^ at a concentration (200 µM) that is known to effectively block Kir2 channels completely abolished the CCh-induced current [66]. Our findings are also consistent with previous reports of muscarinic modulation of Kir2 channels in other brain regions [39], [40], [41], [68], and recent work has also shown that M_1_ receptor activation can inhibit the open probability of Kir2 channels through the depletion of PIP_2_ in a manner similar to muscarinic inhibition of *I*_M_ [39], [58], [60], [61], [69]. It is therefore likely that the M_1_-receptor-dependent depolarization of layer II PaS neurons involve attenuation of both *I*_M_ and *I*_Kir_ via a similar PLCβ-dependent reduction in PIP_2_.

### Functional significance

We have previously reported that layer II of the PaS generates theta-frequency LFP activity in vivo that is dependent on cholinergic mechanisms [11], and we have also shown that layer II PaS neurons display intrinsic voltage-dependent oscillations in membrane potential at theta-frequency that are generated through an interplay between *I*_NaP_ and *I*_h_ [11], [12]. Septal cholinergic projections are known to play an important role in the generation of theta activity in the hippocampal formation [70], [71], and the cholinergic depolarization of PaS neurons observed here likely contributes to the genesis of theta-frequency population activity in the PaS by depolarizing neurons to the subthreshold voltage range at which PaS neurons generate membrane potential oscillations [4], [13], [72]. The hippocampal CA1 region and the anterior thalamus, which contain place-cells and head direction cells, respectively, project to the PaS [5], [6], and many PaS neurons fire in relation to both spatial location and head direction in a manner that is dependent on the phase of ongoing theta activity (“place-by-direction” cells) [1], [2], [73], [74]. This suggests that theta activity in the PaS may modulate the integration of these two complementary spatial inputs, and help determine the timing of the output of the PaS to neurons in the entorhinal cortex.
